# Ag Sinter Bonding to Si Substrate via Temporal Formation and Decomposition of Ag Carboxylate

**DOI:** 10.3390/nano13162292

**Published:** 2023-08-09

**Authors:** Tomoki Matsuda, Rei Kawabata, Takuya Okamoto, Akio Hirose

**Affiliations:** Division of Materials and Manufacturing Science, Graduate School of Engineering, Osaka University, Suita 565-0871, Japanhirose@mapse.eng.osaka-u.ac.jp (A.H.)

**Keywords:** sinter bonding, in situ reaction, silver carboxylate, nanoparticles, silicon

## Abstract

This paper demonstrates the in situ sinter bonding of Ag microparticle pastes to a Si substrate via the temporal formation and decomposition of Ag carboxylate on the surface of Ag microparticles. This was proposed via the investigation of Ag sinter bonding using the redox reaction between Ag_2_O and ethylene glycol, which achieved a bonding strength above 30 MPa even for the bonding temperature at 220 °C. Thermal analysis was used to identify the product of the redox reaction between Ag_2_O and ethylene glycol and determine the bonding temperature because the final reaction facilitates the interfacial sinter bonding with the substrate. Fourier-transform infrared spectroscopy and nuclear magnetic resonance results indicated the in situ formation of Ag salts of carboxylic acids, such as Ag oxalate on the surface of Ag microparticles. Therefore, the sinter bonding process enabled by the in situ formation and subsequent decomposition of these Ag salts was investigated using Ag microparticles and oxalic acid. Observations of the surface and interfacial morphology of the Ag particles after heating revealed the formation of Ag nanoparticles on the surfaces of the microparticles and the formation of sintering necks between the particles. The bonding experiments demonstrated a significant increase in strength with the addition of oxalic acid to the Ag paste due to the enhanced interfacial sinter bonding with the substrate. The in situ formation and decomposition of Ag salts are promising strategies for improving sintered bonds in electronic devices because they can provide enhanced localized sinter bonding using stable insert materials.

## 1. Introduction

Sinter bonding using metal particles is of great interest in electronics packaging to realize superior joint properties, such as heat resistance and high conductivity [1,2]. In particular, wide-bandgap semiconductors such as SiC and GaN require reliable sintering for the development of next-generation power devices. Because metal nanoparticles can lower the temperature for melting and related phenomena [3,4], such as sintering, they have been applied to sinter bonding. However, a metal plating process is performed before sinter bonding [5,6], because direct sinter bonding to nonmetallic materials is generally difficult owing to the differences in their chemical and physical properties. The in situ production of nanoparticles, which can be achieved through the decomposition of compounds, enhance sinter-ability; both nanoparticles and microparticles of metal enable low-temperature sintering via the decomposition of surface compounds [7].

We previously proposed a direct sinter-bonding method for semiconductor materials (e.g., Si and SiC) [8,9] and ceramics (Al_2_O_3_, AlN, and SiO_2_) [10,11] using the in situ generation of Ag nanoparticles during the redox reaction between Ag compounds (e.g., Ag_2_O [12] and Ag carboxylate [13]) and reducing organic solvents (i.e., the polyol reduction process). Such redox reaction-induced sinter bonding involves the formation of an organic residue derived from the reducing organic solvent, which hinders the interfacial sintering of Ag with nonmetallic materials [8]. Thus, the temperature required for Ag sinter bonding to non-metallic materials is influenced by the reaction of the organic residue. It is supposed that lower alcohols, such as reducing organic solvents, are effective for interfacial bonding to nonmetallic materials owing to suppressing the formation of residue near the interface because the thermal decomposition temperature of organic materials is generally dependent on their molecular weight. Lee et al. reported Ag sinter bonding to metals using ethylene glycol to reduce the bonding temperature [14]. Notably, the reaction occurring around the thermal decomposition temperature may be more important for interfacial bonding than the redox reactions occurring at low temperatures, because the organic residue plays a dominant role in the bonding process. Polyols are oxidized to organic acids, such as carboxylic acids, during redox reactions [15]. These carboxylic acids react with Ag to produce Ag carboxylate, which is finally decomposed into Ag [16], hydrocarbons, and CO_2_ upon heating. Therefore, the reaction of Ag carboxylate during the bonding process contributes to interfacial bonding with nonmetallic materials.

In the present study, we investigated lowering the temperature of Ag sinter bonding directly to a Si substrate via a redox reaction using Ag_2_O and ethylene glycol. In addition, the bonding process involving the in situ formation of Ag carboxylate was evaluated based on an analysis of the redox reaction products.

## 2. Materials and Methods

**Figure 1:**
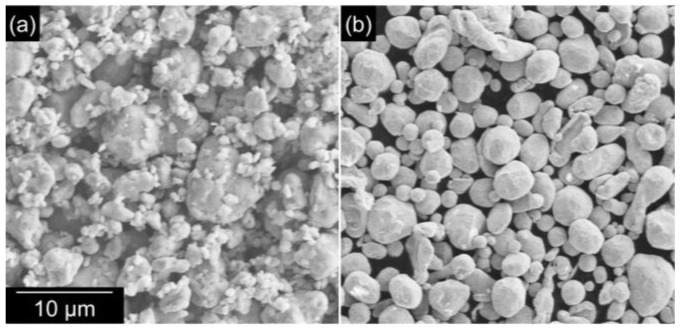
SEM images of (**a**) Ag_2_O and (**b**) Ag particles used as sintering materials.

Commercial Ag_2_O microparticles (Kojundo Chemical Laboratories. Co., Ltd., Saitama, Japan) and Ag particles (diameter of 1.5 μm; Fukuda Metal Foil & Powder Co., Ltd., Kyoto, Japan) were used for sintering experiments. Figure 1 shows field-emission scanning electron microscope (FE-SEM) images of these particles. The Ag_2_O particles were mixed with ethylene glycol (EG) to prepare an Ag_2_O–EG paste (AOE paste). The Ag particles were mixed with an organic solvent composed of oxalic acid and EG to prepare a Ag–carboxylic acid paste (AgC paste); the weight ratio of oxalic acid in the organic solvent was varied from 0 to 40%. Specimens of bare Si chips (4 mm × 4 mm × 1 mm) and metal disks (Ni/Au-plated Cu, 10 mm diameter × 5 mm thickness) were used as substrates to evaluate the joint strength. A 50-μm layer of paste was screen-printed on a metal substrate and dried at 50 °C for 60 min for the AOE paste and at 50 °C for 90 min for the AgC paste. After mounting a Si chip on the paste layer, the samples were heated to the bonding temperature at a heating rate of 60 °C/min and then held for the bonding time at a bonding pressure of 5 MPa in an ambient air atmosphere. The bonding temperature and time were 200–275 °C and 5–60 min, respectively, for the AOE paste and 220–250 °C and 60 min, respectively, for the AgC paste. Subsequently, the joints were cooled under forced air. The joint strength was measured using a die shear test (4000Plus Bond Tester, Nordson DAGE, Aylesbury, UK) at a shear rate of 100 m/s. The microstructures of the particle surfaces and joint cross-sections were observed using FE-SEM (S-4800, Hitachi). The detailed morphology of the AgC particles after heating was observed using a scanning transmission electron microscope (STEM) (Talos F200i, Thermo Fisher Scientific, Waltham, MA, USA) equipped with an energy-dispersive X-ray spectrometer (EDS). To evaluate the reaction behaviors of the pastes, simultaneous thermogravimetry and differential thermal analysis (TG-DTA) measurements (TG8120, Rigaku, Tokyo, Japan) were performed. X-ray diffraction (XRD) measurements (CuKα irradiation) were performed in a Bragg–Brentano geometry to confirm the reduction in Ag_2_O. Fourier-transform infrared spectroscopy (FT-IR) (PerkinElmer Inc., Waltham, MA, USA) and nuclear magnetic resonance spectroscopy (NMR; Bruker, AVANCE NEO 700) were used to evaluate the organic residues of the AOE paste after the redox reaction.

## 3. Results and Discussion

### 3.1. Low-Temperature Decomposition-Induced Sinter Bonding

**Figure 2:**
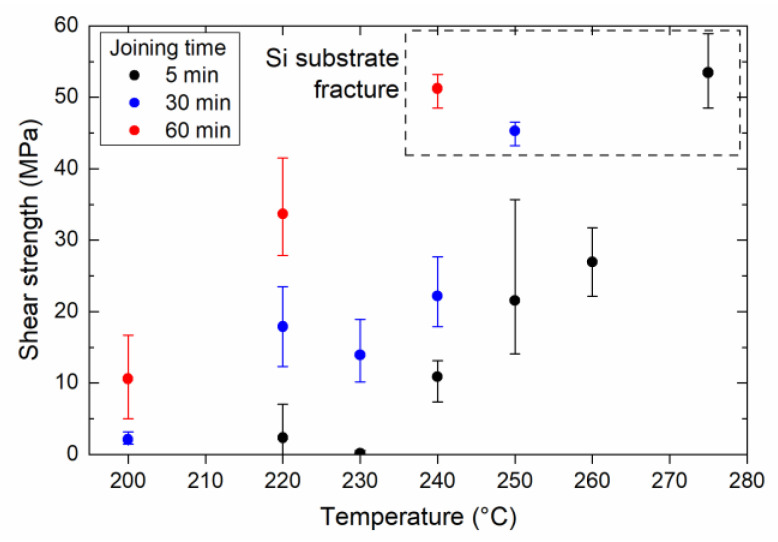
Die shear strength of joints prepared using Ag_2_O–EG paste as a function of bonding temperature and time.

Figure 2 shows the strengths of joints formed using AOE paste obtained by die shear testing. The bonding temperature of 200 °C resulted in a poor joint with a low average strength of 10.6 MPa (and a maximum of 16.7 MPa), even after a bonding time of 60 min. The joint strength increased with increasing bonding temperature and time; in particular, a high-strength joint can be achieved at a lower temperature by increasing the bonding time. For all bonding times, we observed that the Si substrate fractured at a certain bonding temperature, where the die-shear strength exceeded 45 MPa. It is noted that a marked increase in strength occurred around 220 °C.

**Figure 3:**
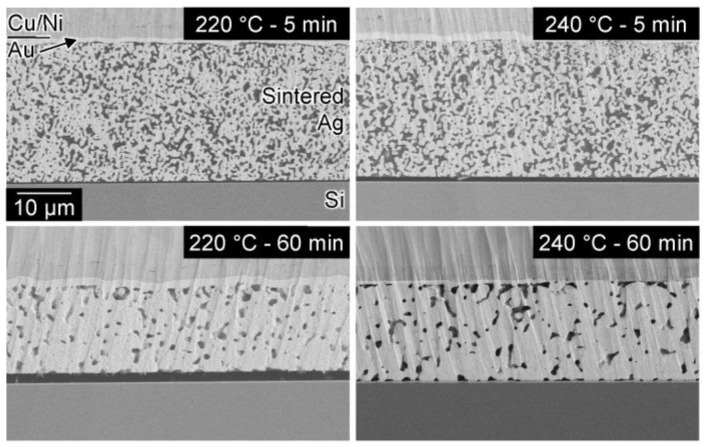
Cross-sectional SEM images of sintered joints prepared using Ag_2_O–EG paste.

The cross-sectional SEM images of the joints (Figure 3) show the microstructural changes in the sintered layer as a function of the bonding temperature and time. It is noted that the peeling of the interface could occur during the sample preparation owing to the low strength of the interface. The porosity of the sintered layer increases with increasing bonding time. However, the porosity did not change significantly with increasing bonding temperature for the same bonding time, although there was an obvious difference in the die shear strength. Therefore, the improved bonding is attributed to the onset of interfacial bonding between the sintered Ag and Si substrate and the subsequent increase in the interfacial bonding area.

**Figure 4:**
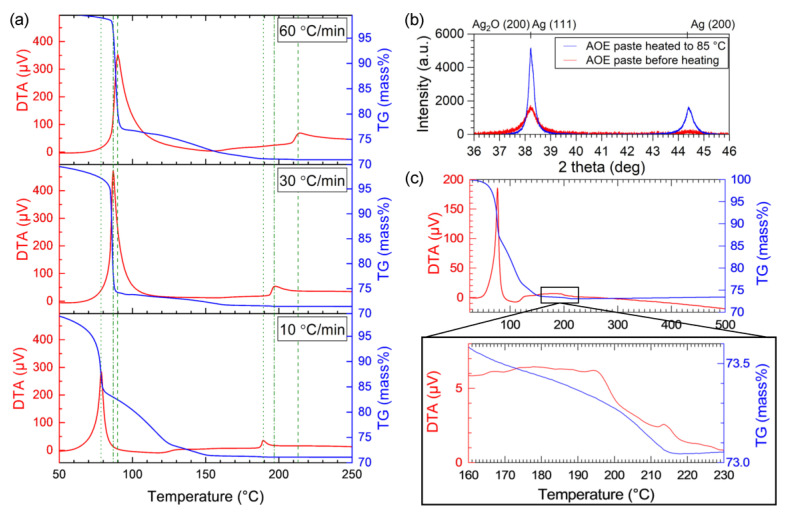
(**a**) TG-DTA curves for Ag_2_O–EG paste as a function of heating rate in air. (**b**) X-ray diffraction result of Ag_2_O–EG paste before and after the heating. (**c**) TG-DTA curves under a N_2_ atmosphere at a heating rate of 10 °C/min.

It is assumed that the onset of interfacial bonding is related to the reaction behavior of the organic solvent because the organic residue prevents interfacial bonding, as described above. To evaluate the reaction behavior of the Ag_2_O paste during heating, TG-DTA analysis was performed on the paste and EG (Figure 4). The exothermic temperature generally shifts to higher temperatures with an increasing heating rate. Hence, the paste was subjected to different heating rates from the bonding temperature to a state close to thermal equilibrium (Figure 4a). The TG-DTA curves for the heating rate used in the bonding experiments (60 °C/min) show exothermic reaction peaks at 90 and 215 °C. The fast reaction with a steep weight decrease above 60 °C is attributed to the redox reaction between Ag_2_O and EG, which was confirmed by XRD measurement on the paste before and after the heating to 85 °C (Figure 4b). The latter reaction began at ~210 °C. This reaction is attributed to the decomposition of the organic residue, because the decomposition of Ag_2_O and evaporation of EG involve endothermic reactions. It is known that protective agents for Ag particles or organic solvents prevent particle sintering [17,18]. Thus, elucidating the decomposition behavior of organic substances is important for understanding their bonding behavior. Although the exothermic reaction temperature decreased with a decreasing heating rate, the exothermic reaction converged to ~190 °C for a heating rate of 10 °C/min. 

TG-DTA analysis under a N_2_ atmosphere was performed on the Ag_2_O paste at a heating rate of 10 °C/min to investigate the influence of the atmosphere (Figure 4c). Even under a N_2_ atmosphere, the redox reaction is characterized by an exothermic peak at 75 °C. The redox reaction between Ag_2_O and EG occurred regardless of the atmosphere. However, the gradual weight loss continued up to 215 °C under the N_2_ atmosphere, as shown in the magnified graph for the temperature region of 170–230 °C; no new reactions occurred at 190 °C compared to those observed in the air. This indicates that the organic residue, which decomposes exothermally at 190 °C under the thermal-equilibrium conditions in air, remains under a N_2_ atmosphere. Furthermore, it is assumed that the organic residue can be removed using a sufficient holding time if the bonding temperature is higher than its decomposition temperature. This explains the relationships between the die shear strength, bonding temperature, and bonding time (Figure 2).

**Figure 5:**
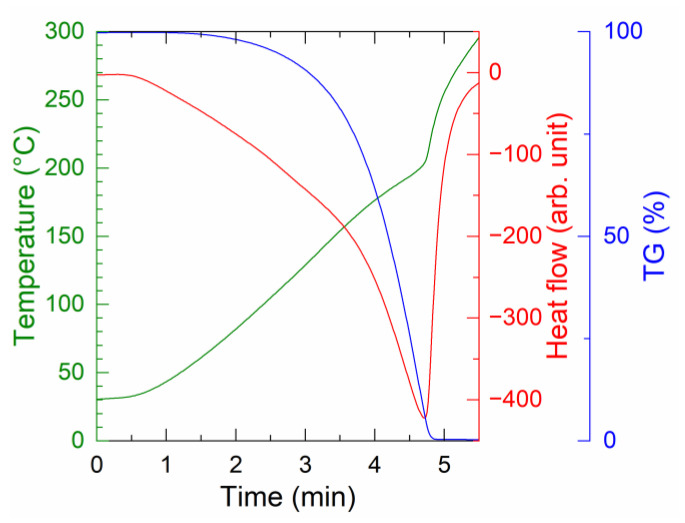
TG-DTA curves of the organic residue extracted from the Ag_2_O–EG paste after the redox reaction.

The organic residue remaining after the redox reaction of the AOE paste was evaluated. A paste was prepared by mixing Ag_2_O and EG (1:2), followed by heating at 150 °C. The organic residue was extracted by centrifugal separation. Figure 5 shows the TG-DTA results for the organic residue measured at a heating rate of 60 °C/min. The organic residue decomposed at approximately 200 °C with an endothermic reaction, unlike the exothermic reaction confirmed for the AOE paste. Therefore, it is proposed that the organic residue decomposed exothermally in the presence of the Ag produced by the redox reaction.

**Figure 6:**
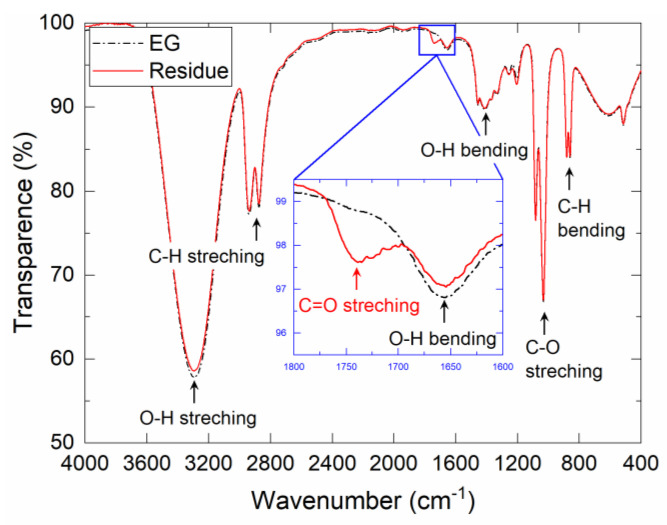
FT-IR spectra of the EG solvent and organic residue extracted from the Ag_2_O–EG paste after the redox reaction.

**Figure 7:**
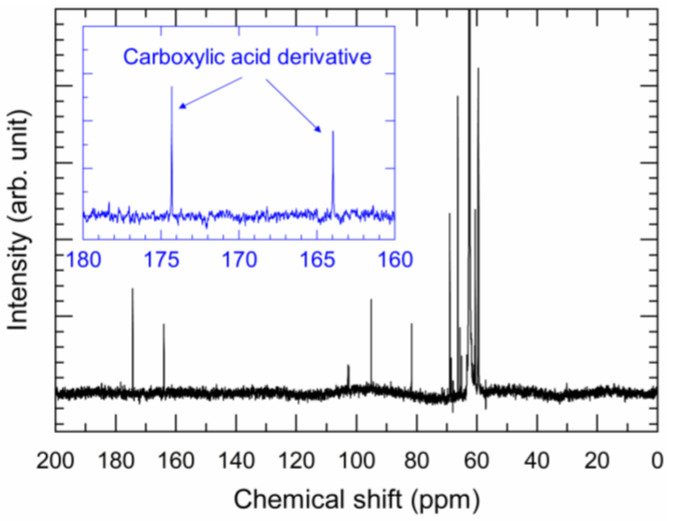
^13^C NMR spectrum acquired for the organic residue extracted from the Ag_2_O–EG paste after the redox reaction.

FT-IR and NMR analyses were performed to investigate the organic residue (Figure 6 and Figure 7). The FT-IR analysis showed that the spectra of the organic residue were very similar to those of EG, indicating that EG was the major component of the organic residue (Figure 6). On the other hand, there was an absorption band at 1700–1770 cm^−1^ that was not observed for EG. It is known that carboxylic acid C=O stretching absorbs IR in this wavenumber range, indicating that the organic residue is composed of EG and carboxylic acid. Furthermore, the ^13^C NMR spectrum shows two absorption peaks at 160–180 ppm, which are attributed to the carboxylic acid derivatives (Figure 7). These chemical shifts are close to those of glycolic acid [19] and oxalic acid [20], which can both be produced by the oxidation of EG. It should be noted that various chemical shift values have been reported in the literature. However, the FT-IR and NMR results indicate the formation of carboxylic acids as oxidation products of EG after the redox reaction between Ag_2_O and EG. 

From these results, it was concluded that the organic solvent decomposed via an exothermic reaction between Ag, carboxylic acid, and oxygen. The reaction between Ag and carboxylic acids has been reported in the reaction of Ag flakes, in which a lubricant containing carboxylic acids, such as fatty acids, is added to prevent particle aggregation [21]. In general, carboxyl groups adsorb onto the Ag surfaces [22,23], and the methyl groups prevent the Ag particles from aggregating. It was confirmed that the carboxylic acids on Ag flakes can decompose via an exothermic reaction during heating under ambient air, whereas they are decomposed without an exothermic reaction in N_2_ [21,24]. These reaction behaviors are consistent with those of the AOE paste after the redox reaction. 

It should be noted that carboxylic acids undergo a decarboxylation reaction in the presence of a metal catalyst [25,26,27,28]. Ag ions are often used as an appropriate catalyst, and it has been reported that Ag is present in ionic form in carboxylic acid solutions and forms Ag salts of carboxylic acid during the reaction process. The decomposition of the Ag salts of carboxylic acid is represented by the following equation [29]:

R-COOAg → Ag^0^ + R^⋅^ + CO_2_ (1)

Here, R is the hydrocarbon side chain, and R^⋅^ is the hydrocarbon radical, which represents the final product change with the type of carbon chain. Thus, the decomposition of carboxylic acid by Ag involves the formation and decomposition of Ag salts of carboxylic acid. There was a difference in the ambient temperature for the exothermic reaction between the Ag flake and the Ag salt of the carboxylic acid. This is because the exothermic reaction of the Ag flakes is attributed to the oxidation of the lubricant layer on the surface, which is composed of Ag and fatty acids [21,24]. In other words, the decomposition of the Ag salt of the carboxylic acid present on the Ag surface could be driven by the oxidation of organic matter via an exothermic reaction in ambient air. Thus, the coexistence of Ag, carboxylic acid, and oxygen results in the formation and decomposition of Ag salts of carboxylic acid. AOE paste may undergo the same reaction route after the redox reaction between Ag_2_O and EG, producing Ag nanoparticles and carboxylic acids as EG oxides. The decomposition of carboxylic acids has been reported to improve the electrical conductivity and sintering ability of Ag [30]. Some studies reported bonding using the decomposition of Ag salts of carboxylic acids to form Ag nanoparticles [13,29,31]; in particular, Ag oxalate enables the direct bonding of Si and SiO_2_ substrates. Therefore, the decomposition of carboxylic acids, which temporarily involves the formation of Ag salts, is considered to play a significant role in the sintering of Ag particles and the formation of dissimilar interfaces. 

### 3.2. Bonding via the Reaction between Ag and Carboxylic Acid

**Figure 8:**
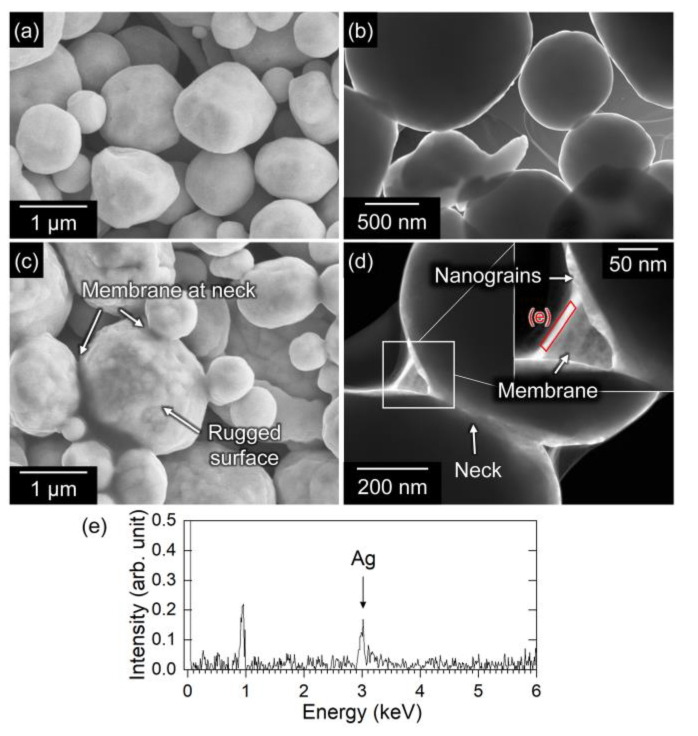
Surface morphology of Ag particles heated to 240 °C. (**a**,**c**) SEM and (**b**,**d**) DF-STEM images of Ag particles mixed with (**a**,**b**) EG or (**c**,**d**) EG-40% oxalic acid. Inset of (**d**) shows the presence of nanograins and neck formation. (**e**) EDS spectrum acquired from the neck between Ag particles shown in (**d**).

The previous results imply that the coexistence of Ag and carboxylic acids can enhance sinter bonding to a nonmetal substrate. To elucidate the bonding mechanism, we investigated the bonding process using Ag microparticles and oxalic acid, which is an oxidation product of EG. Figure 8 shows SEM and dark-field STEM (DF-STEM) images of AgC paste with 0% and 40% oxalic acid after heating to 240 °C. After heating, the Ag microparticles with EG exhibited a smooth surface morphology and no sintering among the particles (Figure 8a,b). In contrast, the SEM image of the AgC paste with 40% oxalic acid shows that the Ag particles have a rough surface and began to sinter locally (Figure 8c). Such sintering behavior is also evidenced by the sintering necks between particles shown in the DF-STEM image, where thin membranes were formed around the neck (Figure 8d). As shown in Figure 8e, EDS spectrum acquired from the neck revealed the presence of Ag; it is noted that a peak at approximately 1 keV would be attributed to background derived from the Cu grid. Furthermore, nanograins were formed on the surface of the Ag microparticles, which correspond to the rough surface features observed by SEM. Therefore, it was confirmed that Ag nanoparticles were formed during heating. These results indicate that the mixture of Ag and carboxylic acid (here, oxalic acid) induces the formation of the Ag salt of carboxylic acid, which subsequently decomposes to newly form nanoscale Ag. These processes play an important role in enhancing sintering ability as nanoscale Ag acts as a sintering aid for particles.

**Figure 9:**
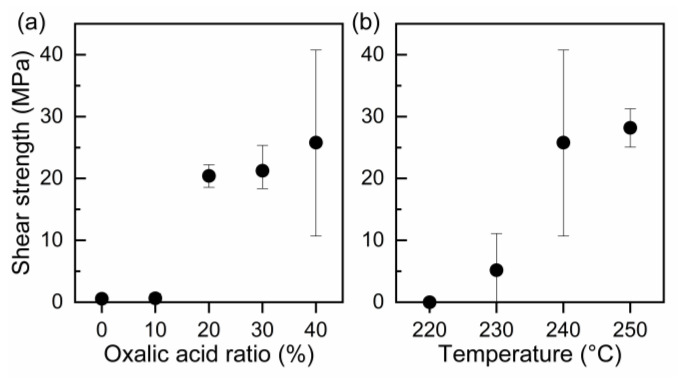
Shear strength of joints as a function of (**a**) oxalic acid ratio (bonded at 240 °C) and (**b**) temperature (Ag–EG-40%OA paste).

**Figure 10:**
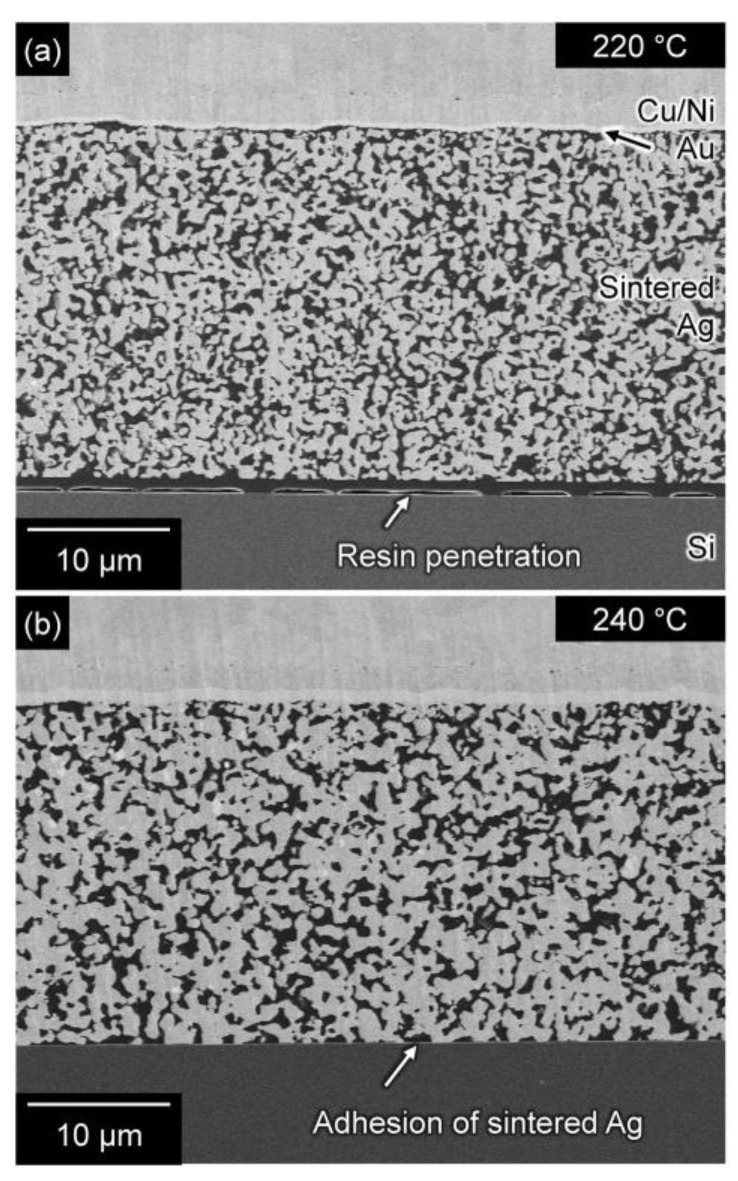
Cross-sectional SEM images of joints bonded at (**a**) 220 °C and (**b**) 240 °C showing the adhesion of sintered Ag to the substrate at 240 °C.

We evaluated the strength of the bond formed by the AgC paste as a function of the ratio of oxalic acid in the paste and the bonding temperature. Figure 9a shows the influence of the oxalic acid content on the shear strength of joints bonded at 240 °C. Notably, the shear strength of the joint prepared using the paste comprising the Ag microparticles and EG was approximately zero. However, the joint strength increased significantly when more than 20% oxalic acid was added, indicating that the addition of oxalic acid assisted the sintering of Ag particles by the generation of Ag nanoparticles on the surface of the original Ag microparticles. Furthermore, Figure 9b shows the influence of the bonding temperature on the shear strength of the joints bonded for 60 min using AgC paste with 40% oxalic acid. Although a low strength was obtained at temperatures up to 220 °C, the strength significantly increased above 230 °C. There was a difference in the onset temperature of the joint strength between the AOE and AgC pastes, which could be due to different reaction processes. The AOE paste initially reacts at low temperatures to form Ag nanoparticles with large surface areas. In contrast, the AgC paste takes longer to react with the Ag microparticles and oxalic acid, owing to its large particle size. Previous research reported that Ag oxalate mixed with EG decomposed at 220 °C and increased the strength of Si/Au joints [13], which is consistent with our results for the AgC pastes. Furthermore, Figure 10 shows the cross-sections of the joints bonded at the temperature above 240 °C drastically enhanced the interfacial bonding between the sintered Ag layer and the Si substrate. Therefore, the AgC paste is thought to bond directly to the Si substrate via the formation of an Ag salt and the subsequent generation of Ag nanoparticles when the salt decomposes at high temperature. 

## 4. Conclusions

The in situ sinter bonding of Ag to a Si substrate using temporal formation and decomposition of Ag carboxylate was demonstrated, and the bonding mechanisms were elucidated. The main findings are summarized as follows:

1. Ag_2_O–EG paste was used to lower the bonding temperature to 220 °C, while simultaneously achieving a bond strength above 30 MPa.
2. TG-DTA showed that the organic residue remaining after the redox reaction between Ag_2_O and EG decomposes at approximately 215 °C, which occurs in the presence of Ag produced by the redox reaction. Spectroscopy analyses indicated the formation of carboxylic acid and Ag salts in the presence of Ag after the redox reaction.
3. Typically, it is not possible to achieve direct sinter bonding to a Si substrate using Ag microparticles. However, the addition of oxalic acid resulted in the formation of Ag nanoparticles on the surface of the original microparticles, which enhanced sintering (evidenced by the observation of necks between the particles). The enhanced sintering achieved by the addition of oxalic acid resulted in a significant increase in the bond strength. Therefore, bonding using the in situ formation and decomposition of Ag salts is a promising process because it can offer localized sinter bonding.

## Data Availability

The datasets generated and analyzed during the current study are available from the corresponding author upon reasonable request.
